# Leaving no one behind in armed conflict-affected settings of Africa: is universal health coverage a possibility or mirage?

**DOI:** 10.1186/s41256-024-00360-3

**Published:** 2024-05-28

**Authors:** Olushayo Oluseun Olu, Amos Petu, Abdulmumini Usman

**Affiliations:** World Health Organization Regional Office for Africa, Brazzaville, Republic of Congo

**Keywords:** Universal health coverage, Sustainable development goals, Conflicted-affected settings, Humanitarian-development divide, Africa

## Abstract

The world is off track six years to the 2030 deadline for attaining the sustainable development goals and universal health coverage. This is particularly evident in Africa’s armed conflict-affected and humanitarian settings, where pervasively weak health systems, extreme poverty and inequitable access to the social dimensions and other determinants of health continue to pose significant challenges to universal health coverage. In this article, we review the key issues and main barriers to universal health coverage in such settings. While our review shows that the current health service delivery and financing models in Africa’s armed conflict-affected settings provide some opportunities to leapfrog progress, others are threats which could hinder the attainment of universal health coverage. We propose four key approaches focused on addressing the barriers to the three pillars of universal health coverage, strengthening public disaster risk management, bridging the humanitarian-development divide, and using health as an enabler of peace and sustainable development as panacea to addressing the universal health coverage challenge in these settings. The principles of health system strengthening, primary health care, equity, the right to health, and gender mainstreaming should underscore the implementation of these approaches. Moving forward, we call for more advocacy, dialogue, and research to better define and adapt these approaches into a realistic package of interventions for attaining universal health coverage in Africa’s armed conflict-affected settings.

## Background

The Sustainable Development Goals (SDGs) are a set of aspirational global development goals underpinned by the principles of equity (leave no one behind), human rights, accountability, and sustainability [1]. The third goal (SDG3) aims to promote well-being and healthy lives for all world citizens and is anchored on the concept of Universal Health Coverage (UHC), which has been designated as sub-goal SDG 3.8. SDG 3.8 aims to achieve “*universal health coverage, including financial risk protection, access to quality essential health-care services and access to safe, effective, quality and affordable essential medicines and vaccines for all”*. UHC ensures that every citizen of a country has access to good quality health services which they require without any financial hardship [2]. Achieving it requires a strong, well-resourced, and functional health system that delivers good quality health care across the life course at all levels. Unfortunately, six years to the 2030 deadline for attaining the SDGs and UHC, the world is off track [3]. While some appreciable progress has been made in the journey towards UHC globally, some regions continue to lag behind. The UHC service coverage index, which is the geometric mean of fourteen tracers of health service coverage and reported on a unitless scale of 0 to 100 [4], is estimated at 48.71 for Africa, and this is far behind other regions. Glaring disparities in progress are evident within the continent, with most low-income African countries remaining below the continental average [5]. This disparity is particularly apparent in armed conflict-affected settings [5].

An armed conflict-affected country is one that has recorded more than ten conflict-related deaths per 100,000 population across many of its regions [6]. Globally, the number of such countries that mostly require international humanitarian assistance has been increasing lately [7], with more than 60% of them located in Africa^1^ [8]. As of 2023, an estimated 40 million Africans are displaced internally or externally mainly due to armed conflicts [9]. The prevailing situation in African countries, particularly the pervasively weak health systems and inequitable access to the social dimensions of human development and other determinants of health, pose significant challenges to UHC. This is further exacerbated by the insecurity, population displacement, loss of livelihoods and extreme poverty, which are hallmarks of armed conflict situations. The UHC service coverage index, in the worst armed conflict-affected countries of Africa such as Somalia, South Sudan, and the Central Africa Republic, were respectively 27.33, 31.83 and 32.46 [4], which are far below the continental and global averages. This is of grave concern given that armed conflict-affected people have peculiar health needs and aspirations^2^ which require UHC. Paradoxically, many of the conflicts are fueled by competition for resources, which, if put to good use, could turn the tide in the progress of these countries towards UHC.

The foregoing brings some critical questions to mind what is the outlook for the achievement of UHC in Africa’s armed conflict-affected countries? Is the paradigm of “leaving no one behind” regarding health a possibility or mirage in these contexts? Given the aspirational nature of the SDGs and UHC, should Africa’s armed conflict-affected countries even strive to attain these goals? d’Harcourt et al. have called for a realistic adaptation of the SDGs to the unique realities of countries [10]. On the other hand, a 2018 call to action on UHC in emergencies asserted that UHC is achievable if there are joint actions to strengthen the humanitarian-development efforts at the country level [11]. We agree with these assertions and posit that as onerous as it is, attainment of UHC is possible in conflict-affected situations if innovative, home-grown, context-specific, and well-planned strategies are used to extend service delivery to the last mile. Nevertheless, we believe that the aspirational SDG and UHC goals would need to be translated into realistic and stepwise targets which could be practically achieved in the remaining six years before the 2030 deadline [10]. While several authors have debated the issue of UHC in humanitarian crisis settings, few have specifically focused their discourse on Africa’s armed conflict-affected settings. Furthermore, only a few have provided explicit and practical guidance on how the barriers to UHC can be specifically surmounted in such settings [12–16]. In this article, we, therefore, review the key issues and main obstacles to UHC in Africa’s armed conflict-affected settings. Based on our field experiences and a review of existing literature^3^, we suggest practical strategies to leapfrog progress towards UHC in such settings. Our suggestions aim to stimulate further dialogue and research on the proposed and newer strategies that could accelerate progress towards UHC in Africa’s armed conflict-affected settings. While the article primarily focuses on chronic armed conflict-affected situations in Africa, its conclusions and recommendations could also be applied to other humanitarian crises elsewhere.

### Do the current emergency health service delivery and financing mechanisms in Africa's armed conflict-affected settings meet the UHC aspirations?

Current emergency health service delivery and financing models in Africa’s armed conflict-affected settings are largely emergency response-focused with few elements of transition, early recovery, and development programming [17]. This humanitarian-development divide often results in chronically weak health systems that cannot deliver UHC [15]. Humanitarian health priorities are primarily determined by humanitarian organisations in a top-down, mainly humanitarian donor-driven manner with little participation of the affected populations and development actors. These health priorities, which are primarily aimed at saving lives, are usually skewed towards addressing the direct impact of conflicts (trauma and injury) and infectious diseases, with little attention to non-communicable diseases apart from mental health and psychosocial care [18]. This is at variance with the UHC approach which encompasses person-centered essential health services, and that goes beyond immediate lifesaving to promoting health. In the face of compromised government institutional capacity for public health governance and weak policy environments, infrastructures, and systems, services are mainly provided by national or international non-governmental organisations contracted by multilateral partners. The service delivery models are mainly health facility-based with pockets of community-based health initiatives, mobile clinics, outreaches, and home visits, which are essential in reaching the last mile [19]. The coordination mechanisms for health in these settings are predominantly humanitarian-based and led by the health cluster that has limited mandate and capacity for coordinating health system recovery and resilience building [20].

The increased demand for healthcare services, a key feature of armed conflicts, usually overwhelms an already disrupted health system, often resulting in poor quality of services. Ramadan et al. identified quality of care as a significant challenge in armed conflict-affected settings and attributed it to inadequate and maldistribution of healthcare staff, insufficient and poor-quality medical equipment, and lack of political commitment and accountability [21]. Anecdotal evidence and our field experiences have also shown that current health service delivery objectives in these settings focus more on increasing access to services irrespective of their quality which negates the UHC principles.

Humanitarian financing requirements have grown more than five-fold in the last couple of years, while funding has only increased three-fold, resulting in an average yearly shortfall of 40% [22]. For example, only 40% of the global humanitarian funding requirement of US$56.7 billion was met in 2023 [23]. Yet a significant proportion of these international funds are allocated to vertical humanitarian programmes with little or no funds for transition and early recovery of systems, including health. Diversion of national resources to fund the defense sector and reduction in revenue generation often results in reduced health financing and increased dependence on donor aid, which is unsustainable in the long term [24]. Based on the humanitarian principles, user fees are usually not charged in Africa’s chronic conflict-affected settings to improve access to health services, which aligns with UHC aspirations [25, 26]. However, our field experiences show that the sustainability of such free services in the face of dwindling domestic healthcare financing and international humanitarian funding remains a critical challenge.

While some of the preceding service delivery and financing models offer some opportunities to leapfrog the attainment of UHC in Africa’s armed conflict-affected settings, others are threats that would hinder progress. This is further compounded by the peculiar features of armed conflict situations, which are barriers to UHC. Wong identified loss of livelihoods, poverty, geographical barriers, mainly due to insecurity and disparities in cultural, religion and gender norms and inequalities as some of the obstacles to the attainment of UHC [27]. These barriers are more so in armed conflict-affected settings where the populations are displaced into camp settings with harsh living conditions and limited access to services. Other authors have pointed to inappropriate health service delivery models [28], high out-of-pocket payments for health services [29], attacks on health systems and infrastructure, existence of dual humanitarian and development contexts, the fragmentation in the response efforts and the dual burden of conflict and natural disasters as other barriers to UHC [12]. Furthermore, accurate measurement of progress towards global development goals is a challenge in Africa’s armed conflict-affected settings. The humanitarian reporting system is often vertical and may not be captured in the calibration of the UHC service coverage index. On the other hand, national health development measurement mechanisms such as demographic and household and mutilple indicator surveys which are used to determine the UHC index often exclude armed conflict-affected settings [10]. The preceding humanitarian-development puzzles create a beneficial incentive of an ad-hoc but less accountable scenario of resource use. This must make way for a steady state development paradigm with its attendant demand for better transparency and equity in resource allocation and use, which is more likely to impact UHC positively.

### How can progress towards UHC be accelerated in conflict-affected settings of Africa?

**Fig. 1:**
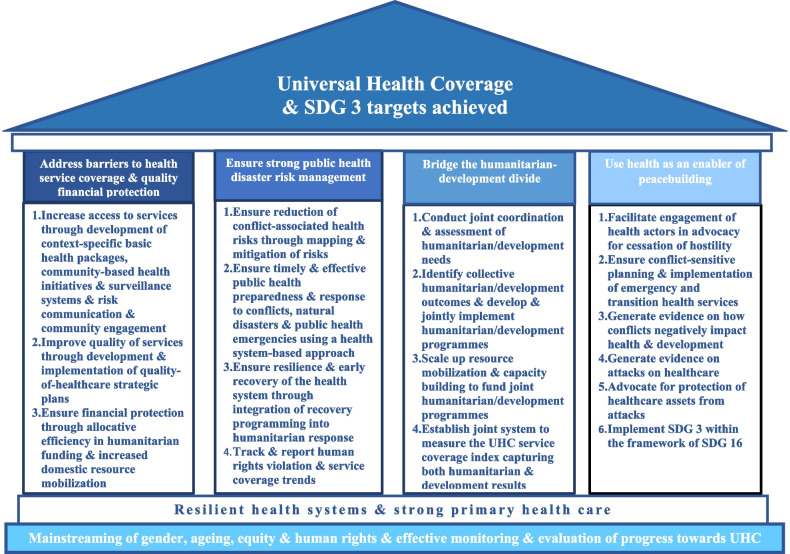
Proposed approach for fast racking attainment of universal health coverage in Africa’s armed conflict-affected settings

We concur with the several authors who have postulated that UHC is possible in armed conflict and humanitarian situations and have proposed general strategies for doing so [12–16, 28]. However, humanitarian health services alone cannot achieve UHC in these settings, thus, we argue that the humanitarian response package must include essential health services package along the life course, as part of the national health development planning and funding. This will provide two advantages. Firstly, the gains towards UHC will be consolidated. Secondly, the transition from humanitarian to more sustainable health development programming will be seamless. This is corroborated by Bernal et al. and Devkota et al. who described the critical success factors for good public health outcomes in the Colombian and Nepalese armed conflicts [30, 31]. These include among others, the prioritization of disadvantaged groups such as women, children and the aged, use of community-based health initiatives, implementation of conflict-sensitive development programmes and health policies which are tailored to local needs. Therefore, we affirm that more concerted and organised efforts are required to design and implement innovative and practical strategies for strengthening health systems, ensuring the quality of care and financial protection, and protecting health assets in Africa’s armed conflict-affected settings. Based on the existing literature, our field experiences, and the foregoing discourse, we propose a four-pronged approach for accelerating progress towards UHC in Africa's conflict-affected settings (Fig. 1). These are to: i) address the barriers to the three pillars of UHC, ii) strengthen health security and public health disaster risk management using a health system strengthening approach, iii) bridge the humanitarian-development divide, and iv) use health as an enabler of peacebuilding and sustainable development.

### Addressing the barriers to service coverage, quality of care and financial protection

In conflict-affected contexts, improving access to a full complement of health services can be viewed from the scope of available services and diffusion in geographic access to those services. First, ensuring an appropriate scope of health services requires evidence-informed and context-specific determination of cost-effective basic packages of health services, which address the major causes of morbidity and mortality and have the highest impact [32]. Planning and implementing such packages in a manner that prioritizes the most vulnerable persons, such as children, women and aged persons, and addresses the gender, social, cultural, and religious disparities will further improve access to healthcare. Second, we advocate for more significant investments in community-based health initiatives such as community health workers [33, 34] to address the geographical barriers to health services in armed conflict-affected settings [35]. This is particularly important in bringing vital preventive services, such as vaccination, integrated management of childhood illnesses, antenatal and postnatal care closer to armed conflict-affected persons. Community-based surveillance has also proven useful in rapidly detecting and responding to disease outbreaks, monitoring the public health trends among conflict-affected populations, and providing evidence for determining the basic essential package of health services [36]. The successful use of mobile medical teams to deliver health services in such settings has also been documented and is recommended where feasible [37]. Prioritisation of community mobilisation and participation as an integral component of community-based health initiatives through the introduction of risk communication, community engagement, health promotion, and preventive interventions are also critical in bringing preventive services closer to affected populations.

In armed conflict situations, good quality health services are critical for ensuring good public health outcomes. This could be achieved in several ways. First, we propose developing and implementing health service quality strategic plans as integral components of humanitarian health action and health sector strategic plans [38]. Such strategic plans should define minimum quality standards for both private and public providers of emergency healthcare services and the benchmarks for supervising, monitoring, and evaluating them [39]. Second, we recommend strengthening the oversight, governance, and accountability functions of national governments and humanitarian coordination mechanisms concerning the quality of care. Third, we call for training emergency healthcare workers on how to monitor and improve healthcare quality as an integral part of emergency preparedness and response capacity building. Fourth, recognising the limited technological infrastructural and skills in conflict-affected settings, digital health technologies may also provide opportunities to expand access to health services and improve healthcare quality [40, 41].

Ensuring financial protection of armed conflict-affected populations requires a two-pronged approach that guarantees adequate financing of good quality healthcare on the one hand, while removing the financial burden on the affected populations on the other. The possible strategies to achieve these in Africa’s armed conflict-affected settings include reducing inefficiencies in the allocation of humanitarian funding, which we believe would free up more funds to cover more services and improve the quality of the existing ones [42]. More attention to achieving technical efficiency could trigger a drive towards allocative efficiency with the maximum utility of resources that generally pour into Africa’s conflict-affected areas. For instance, this will settle the argument of campaign versus routine service delivery mode in chronic conflict settings. Second, we advocate for implementing sustainable strategies to mobilise additional domestic resources through corporate taxation and corporate social responsibility of the private sector [43] to supplement domestic funding of healthcare services where possible [43]. For example, Hannah et al. demonstrated the feasibility of corporate taxation in advancing the SDG agenda in six countries, including the Democratic Republic of Congo, which is armed conflict-affected [43]. We believe that similar strategies could be used to broaden the tax net from the mineral-rich industries in conflict-affected countries such as South Sudan, Central African Republic, Niger etc., which could increase domestic financing of health. Third, Jowett et al. also highlighted the importance of pooling humanitarian and development funding to scale up service delivery and improve efficiencies [44]. These could also support pooled procurement of health commodities and contracting health services with significant cost-savings and improved access to health services. Fourth, we believe that community cooperative societies could boost livelihoods and the ability of conflict-affected populations to participate in community-based health insurance schemes, particularly in relatively stable situations such as internally displaced persons camps. Community-based health insurance schemes could also improve access to more specialised care, which may not be included in the essential package of healthcare services. Fifth, public-private partnerships for health services delivery in conflict-affected areas are also imperative. Private providers financed through community-based insurance schemes could complement the humanitarian health services delivery, thus expanding the scope of care. Jowett et al. have also suggested using cash and voucher assistance to cover the indirect cost of accessing healthcare, such as transportation [44]. We believe this could also directly improve access to specialised healthcare services.

### Strengthening public health disaster risk management

UHC and global health security are frequently referred to as *“two sides of the same coin”*[45]. Thus, both concepts should be addressed jointly using a health system-strengthening approach [46]. Resolution 64.10 of the World Health Assembly urged countries to incorporate public health disaster risk management (DRM) programmes into national health systems [47]. In this regard, first, strengthening public health disaster risk reduction, preparedness, response, and early recovery capacities as means of protecting and ensuring the integrity of public health systems are imperative in Africa’s conflict-affected settings. This should involve detailed assessment and mapping of the risks that conflicts pose to health systems and the institution of preventive and preparedness interventions to mitigate such risks. Second, a health system-based approach to emergency preparedness and response is also needed to facilitate robust management of the public health risks and consequences of conflicts. Third, integration of health in transition and early recovery interventions into humanitarian response programming is required to ensure that disrupted health systems are systematically recovered from the impact of conflict and built back better [48]. Fourth, establishing early warning and surveillance systems to track human rights violations, attacks on healthcare systems and public health trends could also contribute to accelerating progress towards UHC.

### Bridging the humanitarian-development divide

The United Nations General Assembly recognised the pervasive humanitarian-development divide as one of the critical challenges to achieving good outcomes in conflict situations [49]. Through its Principals, the Assembly committed itself and global stakeholders to bridge this divide by creating a nexus that brings humanitarian and development actors closer to achieving collective outcomes [50]. To operationalise this commitment within the health sector, WHO, in a 2021 guidance document, proposed the establishment of mechanisms for joint coordination, assessment, identification of collective outcomes, development, implementation, monitoring and evaluation of joint plans by both humanitarian and development actors in the health sector of conflict-affected areas [51]. We support these proposals and believe that their implementation could build solid foundations for health system resilience and UHC in Africa’s conflict-affected settings. We call for integrating these proposals into all humanitarian planning, response, and financing mechanisms and training of humanitarian and development actors to practically apply them. Furthermore, we advocate for the establishment of more realistic systems for the measurement of the UHC index in Africa’s armed conflict-affected settings that captures information from both humanitarian and develoipment reporting streams and reflect the complexities in funding and service delivery in such settings.

### Using health as an enabler of peacebuilding

SDG 16 recognises the need to resolve conflicts, promote peace, and engage societies to foster sustainable development [1]. Sustainable development, particularly inclusive access to health and its social determinants, could prevent conflicts and foster peacebuilding [52]. The concept of using public health as an enabler of peacebuilding has gained ground recently [53–56]. MacQueen et al. aptly described the interphase between peace and health and proposed various mechanisms for using health for conflict resolution and peacebuilding [57]. We agree with these mechanisms and believe they will contribute to peacebuilding towards attaining UHC in Africa’s armed conflict-affected settings. We therefore call for better engagement of Africa’s health actors in peacebuilding through advocacy for cessation of hostility, so that vital healthcare care can be delivered to conflict-affected populations. For instance, at the height of the war in El Salvador, the United Nations and the Catholic Church negotiated and secured 3 days of peace every year from 1985 to 1991 during which vital health services including vaccination were delivered to armed conflict-affected populations [58]. Similar strategies were also used in Afghanistan, Somalia and South Sudan to improve polio vaccination coverage [59] and in the emergency response to the COVID-19 pandemic [60]. Furthermore, we call for the generation of evidence on how conflicts negatively impact health and development and advocate for the protection of healthcare assets, particularly health workers, equipment, and supplies, from attacks [61]. In this regard, direct engagement with and advocacy to the warring groups to guarantee the safety and security of healthcare assets is critical [62]. We advocate for conflict-sensitive planning, implementation, monitoring and evaluation of emergency and transition health services in such a way that they contribute to peacebuilding and, ultimately, UHC. Finally, SDG 3 and UHC interventions should be implemented within the broader framework of the other SDGs particularly SDG 16.

## Conclusions

A few years to the global deadline for attaining the SDGs and UHC, the world, particularly Africa, is off track. This is more evident in Africa’s armed conflict-affected settings where the prevailing humanitarian environment and weak capacities pose significant obstacles to UHC. Ensuring that the more than 40 million people displaced by conflicts in Africa are not left behind in the race towards the SDGs and UHC is a moral imperative, which requires the attention of global and African health policymakers, public health practitioners, and researchers. Concerted efforts are therefore required to leapfrog progress towards UHC in such settings in the spirit of “leaving no one behind”. In this regard, we believe that realistic and stepwise approaches to the SDGs and UHC and systematic implementation of the practical, context-specific, and sustainable approaches, which we have described in this article, could fast track progress towards the SDGs and UHC. Implementing these approaches would require addressing practical challenges, such as the humanitarian-centric and donor-driven approaches to healthcare planning and delivery, changing the mindset of humanitarians who focus mainly on implementing short-term lifesaving emergency projects to the detriment of medium to long term health recovery programmes and increasing the health system recovery and development funding in Africa’s armed conflict situations. Furthermore, the implementation of these approaches should be underscored by the principles of health system strengthening and resilience, strong primary health care, equity, the right to health, and gender mainstreaming. Finally, we call for more advocacy, dialogue, and research to better define and adapt these strategies into a realistic package of interventions for attaining UHC in Africa’s armed conflict-affected settings. Specifically, we call for an African regional health and emergency expert consultation to thoroughly discuss and crystalize the key issues and challenges and define the policy, strategic, operational and research shifts which are required for fast tracking progress towards to the SDGs and UHC in Africa’s armed conflict settings.

## Data Availability

Not applicable.

## Abbreviations

DRM: Disaster Risk Management
PHC: Primary Health Care
SDGs: Sustainable Development Goals
UHC: Universal Health Coverage
WHO: World Health Organization

## Footnotes

1 According to the World Bank, the armed conflict affected countries of Africa are Burkina Faso, Cameroon, Central Africa Republic, Democratic Republic of Congo, Ethiopia, Mali, Mozambique, Niger, Nigeria, Somalia, South Sudan and Sudan. According to the international humanitarian law definitions, all these conflicts are non-international armed conflict between States and non-State armed groups.

2 The peculiar health needs of Africa’s armed conflict-affected populations include trauma care, prevention of infectious diseases such as malaria, tuberculosis, HIV and neglected tropical diseases, epidemic preparedness and response, management of malnutrition, mental health and psychosocial care, management of chronic illnesses such as hypertension, diabetes etc.

3 Literature for this perspective was obtained through general searches in the main databases such as Pubmed, Google Scholar, African Journals Online, and African Index Medicus, using the search terms “UHC” OR “universal health coverage” OR “universal coverage” OR “healthcare delivery” AND “armed conflicts” OR “armed conflict” OR “war” OR “wars” AND “Africa” OR “conflict-affected countries”.
